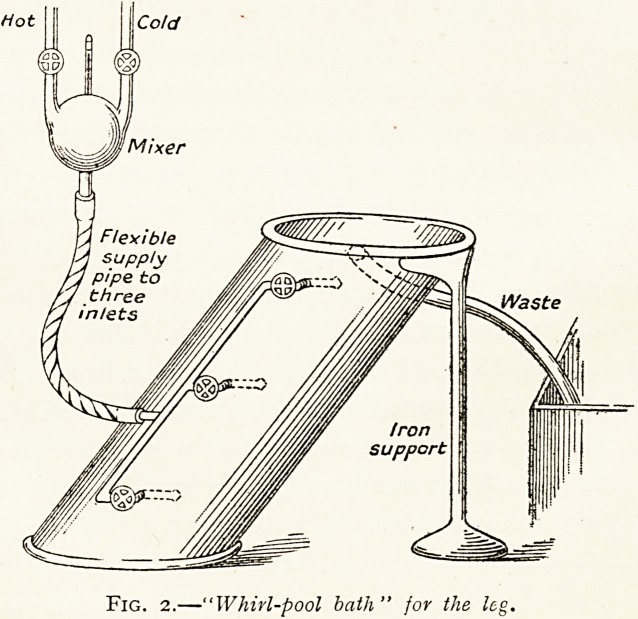# The Reduction of Disabilities from Wounds in War

**Published:** 1916-07

**Authors:** W. Gordon

**Affiliations:** Physician to the Royal Devon and Exeter Hospital


The Bristol

Medico-Chirurgical Journal.

" *Scire est nescire, nisi id me
Scire alius sciret.*"

JULY, 1916.

### THE REDUCTION OF DISABILITIES FROM WOUNDS IN WAR.

BY

W. GORDON, M.D., F.R.C.P.,

*Physician to the Royal Devon and Exeter Hospital.*

THE reduction of disabilities entailed by wounds in war is a matter of national concern. Quite apart from the natural promptings of humanity, gratitude or affection, an urgent problem is presented to us carrying with it consequences of the highest national importance, military, financial and social. We have to consider, as a nation, *and soon*, by what methods of treatment we may in the fullest degree :—

(*a*) hasten the return of wounded to their units at the front, where every man is needed who can be made sufficiently fit ;

(*b*) reduce the heavy and enduring expense to the country entailed by disabilities, which timely measures may materially lessen ;

(*c*) reduce the impairment of civil industry after the war, occasioned by the numbers of seriously and permanently crippled men.

France has set us an example in grappling with this problem by initiating a system of treatment which is proving a remarkable success. For :—

(*a*) 80 per cent. of the cases, which have undergone a complete course, are returning to their units, the duration of a " good case " being perhaps one to two months, and the average reduction of disability for all cases being 20 per cent. to 30 per cent. ;

(*b*) a remarkable financial saving has been effected for the State in pensions and gratuities by reduction of the disabilities which entail these ;

(*c*) and obviously, according to the measures of (*a*) and (*b*), the problem of impairment of civil industry is being solved in the best manner possible.

That this system is one of great value we have the most convincing evidence. The officers entrusted with its supervision are unanimous in claiming for it an efficacy in the reduction of war disabilities which no other method possesses, and their claims are substantiated by the extension of the system throughout the country. At a time when economy and efficiency are vital national needs, public authorities do not extend their undertakings for visionary advantages. The Grand Palais installation in Paris began its experiment by treating 200 patients a day, now 650 men are being daily treated in the electrical department alone ; in and round Paris other installations have been set up, and in the French Provinces there are already seven fully-equipped and satisfactory institutions of the same sort, as well as four which are less complete.

England cannot claim, so far, to have profited to any considerable extent by the initiative of her Ally in this matter. Thanks to the energy and advocacy of Dr. Fortescue Fox and the Special Committee appointed last year by the Balneological and Climatological Section of the Royal Society of Medicine, a beginning has been made in London, by the establishment of a Physical Clinic for wounded and disabled officers. Also Major Mackenzie has been carrying out an admirable course of treatment for the N.C.O.'s and men, in the Command Depôt at Heaton Park. But, although generally throughout the country massage, electrical treatment and, in some cases, baths, and mechanical apparatus are now being employed, physical treatment *in a "combined" and systematic form* remains still to seek. This is the case, not only as regards officers, non-commissioned officers and men, but also for the large numbers of disabled men discharged from the army. The relief of the disability of the discharged soldier, although he no longer counts from the military point of view, is an economic problem of national importance.

Perhaps the appeal—surely a reasonable one—to give the Grand Palais system at least a fair trial in this country, has not yet reached those departments of the State which it most enduringly concerns. To those Government Offices which will presently have to deal with an unexampled number of war-crippled men, for whom public opinion will rightly demand adequate financial treatment, whilst it will equally rightly denounce such extra expense as might have been prevented, and to those Ministers of State who will have to grapple, after the war, with unnecessarily hampered industries, the problem of the best mode of dealing now with these disabilities can scarcely be considered negligible. Undoubtedly to the public (for the moment unaware of better or worse in the means being taken to care for our wounded) the question will ere long have to be answered—why the methods found so successful in France have not been adequately tried in England.

I will endeavour briefly to describe the system being carried out at the Grand Palais and its Annexe in Paris, as I saw it five months ago, and to outline its results. It will not, I hope, be imagined that I have left my information unreported for that great space of time.

The system (*and it must be carefully observed that it is a system, completely and regularly carried out under skilled and constant supervision*) in its essentials consists of, broadly speaking :—

(1) a preparation by heat, moist or dry;

(2) a thorough course of massage, manipulation, mechanical treatment and electricity;

(3) with, finally, a re-education of the affected muscles by exercises and training;

(4) the results of the whole being checked by careful measurement of initial defect and ultimate gain.

Taking these component elements in order :—

*Preparatory Heat.*—I venture to doubt whether the profession at large in this country has the least idea how profoundly the results of massage may be modified by preparatory heat. My own experience leads me to attach the highest importance to it. I well remember the first case which claimed my attention many years ago. A girl with double hydrarthrosis had been put by me on back splints and Scott's dressing, with disappearance of effusion and fixation of both knees; she was seen by an able surgeon, who advised the breaking down under an anæsthetic of the recent adhesions. Acting on other advice, I packed the knees in hot dry sand daily for twenty minutes, with manipulation, and in a week had the satisfaction of seeing their mobility completely restored. Those were recent adhesions. The following illustrates the use of hot sand on adhesions of some standing, in just the sort of case for which I am advocating this French system. A soldier, whose arm had been torn by a shell, had rigid painful fingers which could not bear much massage or manipulation, so that little progress was being made under excellent surgical supervision. I advised baking the hand daily in hot dry sand, as a preliminary to massage and movement ; the massage became at once much less painful, the joints more supple, and progress much more rapid ; ultimately a very considerable degree of useful mobility was regained.

It cannot be too much emphasised how important it is that such treatment should be adopted early. Dr. Jean Camus, who is the responsible head of the treatment at the Grand Palais, speaks in his last report (*Le Corps de Réeducation Physique du Grand Palais*, Paris, 1916) of the "deplorable" consequences of delay. Had the second of the two cases just quoted been similarly dealt with some months earlier, directly his wounds were healed, much more could probably have been done for him. As soon as the wound is healed this system of physical therapeutics ought to be begun.

*Heat* is applied at the Grand Palais either dry or wet.

(i) *Dry.*—By means of *radiant light and heat,* or in other ways. Anyone who watches the effects of radiant light and heat as a preparation for massage, can testify to their great value in reducing tenderness and hastening progress.

(ii) *Wet.*—The chief means of applying this is the "*eau courante*" or "whirlpool bath" (Figs. 1 and 2),[1] described in the *Lancet* of February 5th. The *eau courante* bath consists of a simple arm, leg or foot bath, in which two or more jets of entry, turned in different directions parallel to the sides, keep the water in a constant state of rapid movement. The sensation to the immersed skin is just like that of being in rapid running water. The temperature is kept about 115° Fahrenheit, and the limb is placed in it for about twenty

[1] These figures have been kindly lent by Dr. Fortescue Fox.

FIG. 1.—"*Whirl-pool bath*" *for the arm.*

FIG. 2.—"*Whirl-pool bath*" *for the leg.*

minutes daily. Whatever the mode of action may be (some think a gentle surface massage is produced), those who have abundant experience assure me that, generally speaking, as a preparation for massage and manipulation, this method is unrivalled. This bath, which has been used in France for some time, is new to this country, and obviously calls for investigation. I have just said "generally speaking," because I am told that some cases do better with dry heat than with wet heat; no means apparently exist by which such cases can be identified at sight, the only way of making sure being to try both methods.

*Massage and Manipulation.*—No one is likely to question the need for these well-established measures. But two points will bear mention. First, massage and manipulation of painful fingers and hands can be much less painfully and much more effectually carried out actually under radiant light and heat. Second, that every month which is allowed to elapse, after the wound has healed, before these measures are adopted reduces the improvement possible.

*Mechanotherapy.*—The Zander method of treatment by mechanical appliances is little known in England, although one or two excellent installations exist. The principles which underlie it are:—

(*a*) That, by isolating, for treatment, just the muscles or groups of muscles which need it, and putting the others at rest, the maximum of benefit is gained with the minimum of fatigue.

(*b*) That, by means of movable weights, the desirable degree of effort can be adjusted exactly.

Incidentally the initial defect and the ultimate improvement can be precisely measured.

The instruments are "active" and "passive." With the "active" the patient uses his muscles to make the movements. With the "passive" the movements are made for him, without any effort on his part. In using these machines it is essential that the patients should be under constant skilled supervision. It is computed that one trained nurse should be allotted to every three machines. Zander's original instruments were exceedingly expensive. Simpler instruments such as those figured in the *Lancet* of March 25th are, however, equally efficient and considerably less costly. A complete apparatus in twelve pieces has been recently designed in France for the treatment of military cases, and the first series of this kind that has reached England has been installed in the London Physical Clinic. They are very similar to those in use at the Grand Palais. The Annexe of the Grand Palais, which is used chiefly for officers, is actually the Paris Zander Institute; it contains about seventy different instruments of the original pattern. At Enghien there is a similar installation to that at the Grand Palais. At Versailles I was shown an ingenious and inexpensive set of instruments from which good results are obtained.

*Electricity* seems to be used in Paris as we use it in England. It forms naturally an important part of the system.

Other methods, such as Bier's treatment, are also used when thought desirable.

*Re-education of Muscles.*—Great stress is laid on this last part of the system. A huge gymnasium is attached to the Grand Palais in which exercises and training of movements are carried out under the supervision of skilled instructors. Three separate corps have been constituted, viz. for upper limb, lower limb and trunk disabilities respectively. It has been found necessary in many cases actually to re-educate the damaged muscles in their restored functions.

*Mensuration* becomes essentially a part of any system which aims not merely at maximum reduction of disabilities, but at fair compensation for disability acquired in the defence of the country. In France there is a regular scale of pensions and gratuities for various degrees of disability from wounds.

At the Grand Palais and other Institutions across the Channel careful measurements of defects are made on the entry of every patient, and carefully compared with the defects on exit. The difference is assessed as "gains," the financial value of which can, according to the scale used, be exactly stated. Of these "gains" I shall speak in a moment.

*Detection of Malingering.*—Dr. Camus has invented an ingenious instrument, whereby malingering can be detected in whatever muscles their pretended disability is seated. The tremor of the voluntary movement of the defective muscle is traced on a revolving drum, and the tracing examined. Its characters distinguish true from false.

*Results at the Grand Palais.*—Dr. Camus's excellent report above referred to brings the results, physical and financial, of the Grand Palais system up to the end of last February.

In six months 3,348 complete treatments have been carried out. Of this number of wounded men, 2,676 (80 per cent.) have been *cured* and sent to their depôts as fit, 457 recommended for the auxiliary services, and 215 discharged.

The reduction of incapacity for work has been carefully noted for every case. This reduction has, on the average, varied from 20 to 30 per cent.

As to the financial saving, it is calculated that the capital value of the gratuities and pensions saved by this reduction of disabilities for the month of February was as follows :—

| For | Capital in francs. |
|---|---|
| 486 Soldiers cured .. .. .. .. .. .. | 2,916,000 |
| 81 Corporals cured .. .. .. .. .. .. | 567,000 |
| 75 other N.C.O.'s cured .. .. .. .. .. | 600,000 |
| 104 Soldiers proposed for Auxiliary Forces | 416,000 |
| 12 Corporals proposed for Auxiliary Forces | 56,160 |
| 14 other N.C.O.'s proposed for Auxiliary Forces .. .. .. .. .. .. .. | 75,040 |
| 75 Soldiers recommended for discharge | 300,000 |
| 9 Corporals recommended for discharge | 21,240 |
| 13 other N.C.O.'s recommended for discharge .. .. .. .. .. .. | 34,840 |
| Total gains for one month .. | 4,986,280 |

The total saving for six months (September to February) is estimated at F. 16,667,720 (at the Grand Palais alone). The rate of reduction has lately increased, and the saving at the rate attained when I saw Dr. Camus in February, if it went on—and there was no reason imaginable why it should not—would amount, he told me, to F. 50,000,000 a year, a saving so great as to render insignificant the moderate initial outlay and upkeep.

It is this *remarkable financial saving* which should commend itself to the attention of our Treasury, *whilst it is attainable.* No doubt, in our country, physical methods, however incompletely applied, have sometimes effected considerable economy. But what I would urge is that the introduction of specialised methods (particularly of baths and mechanical apparatus) and a system such as that adopted in France, would probably secure a much greater reduction of disability—a result which humanity and gratitude alone should make us anxious to achieve—and would also incidentally produce a great decrease of the burden which adequate pensions will place for years on the shoulders of the taxpayer.

The medical services of our Empire (under which title I

would include its entire medical profession) have played a part in this war of which every member of them may well be proud. One would be sorry to see them fall short of the very best, even in a single particular.

I would once more express my cordial thanks to Dr. Quiserne and Dr. Camus, the Medical Officers supervising the Annexe and the Grand Palais respectively, for the unwearying kindness which showed me, whilst I was in Paris, everything that time permitted, and for the valuable reports and illustrations which they have placed at my disposal. Also to my friend Dr. Fortescue Fox for many detailed observations and for his great help in enabling me to obtain the necessary permission from the French Government to observe the methods employed in this department of their Army Medical Work.

---

## Figures and Tables

**Fig. 1. f1:**
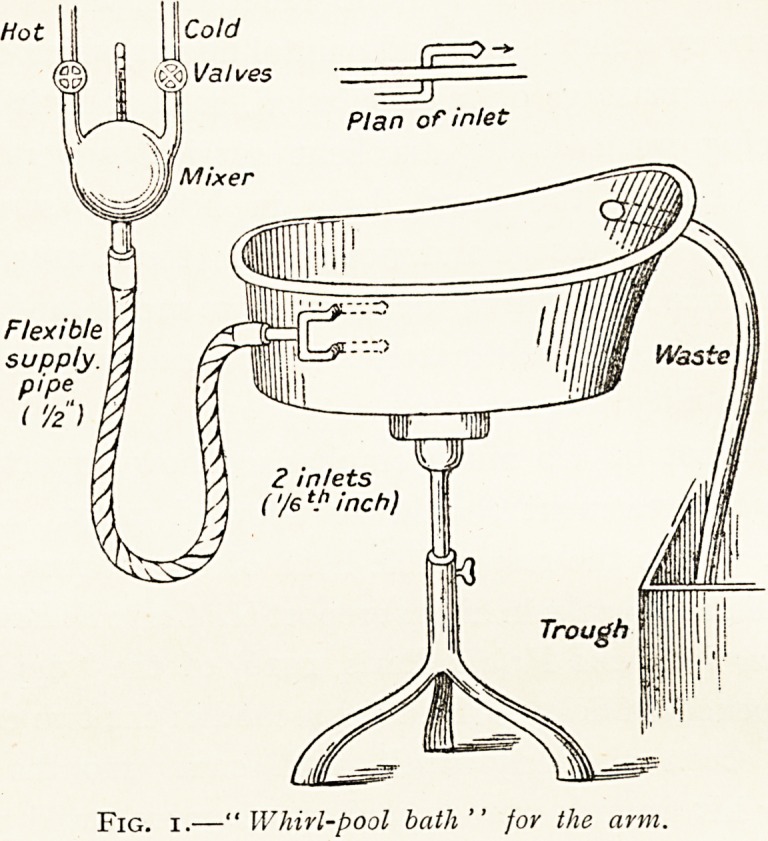


**Fig. 2. f2:**